# Progress on quantum dot photocatalysts for biomass valorization

**DOI:** 10.1002/EXP.20220169

**Published:** 2023-10-02

**Authors:** Weijing Cao, Wenjun Zhang, Lin Dong, Zhuang Ma, Jingsan Xu, Xiaoli Gu, Zupeng Chen

**Affiliations:** ^1^ Jiangsu Co‐Innovation Center of Efficient Processing and Utilization of Forest Resources International Innovation Center for Forest Chemicals and Materials College of Chemical Engineering Nanjing Forestry University Nanjing China; ^2^ Leibniz‐Institut für Katalyse e.V. Rostock Germany; ^3^ School of Chemistry and Physics and Centre for Materials Science Queensland University of Technology Brisbane Queensland Australia

**Keywords:** biomass, carbon neutrality, photocatalysis, quantum dot, renewable resource

## Abstract

Biomass with abundant reproducible carbon resource holds great promise as an intriguing substitute for fossil fuels in the manufacture of high‐value‐added chemicals and fuels. Photocatalytic biomass valorization using inexhaustible solar energy enables to accurately break desired chemical bonds or selectively functionalize particular groups, thus emerging as an extremely creative and low carbon cost strategy for relieving the dilemma of the global energy. Quantum dots (QDs) are an outstandingly dynamic class of semiconductor photocatalysts because of their unique properties, which have achieved significant successes in various photocatalytic applications including biomass valorization. In this review, the current development rational design for QDs photocatalytic biomass valorization effectively is highlighted, focusing on the principles of tuning their particle size, structure, and surface properties, with special emphasis on the effect of the ligands for selectively broken chemical bonds (C─O, C─C) of biomass. Finally, the present issues and possibilities within that exciting field are described.

## Graphical abstract

Quantum dots (QDs) catalyzed biomass valorization into high‐value‐added chemicals and fuels under solar light is an essential measure to achieve the target of carbon neutrality. This review summarizes the current development of the rational design of QDs photocatalysts for efficient biomass valorization including tuning particle size, structure, surface properties, and ligands.

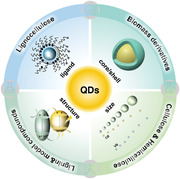

## 1 INTRODUCTION

**FIGURE 1:**
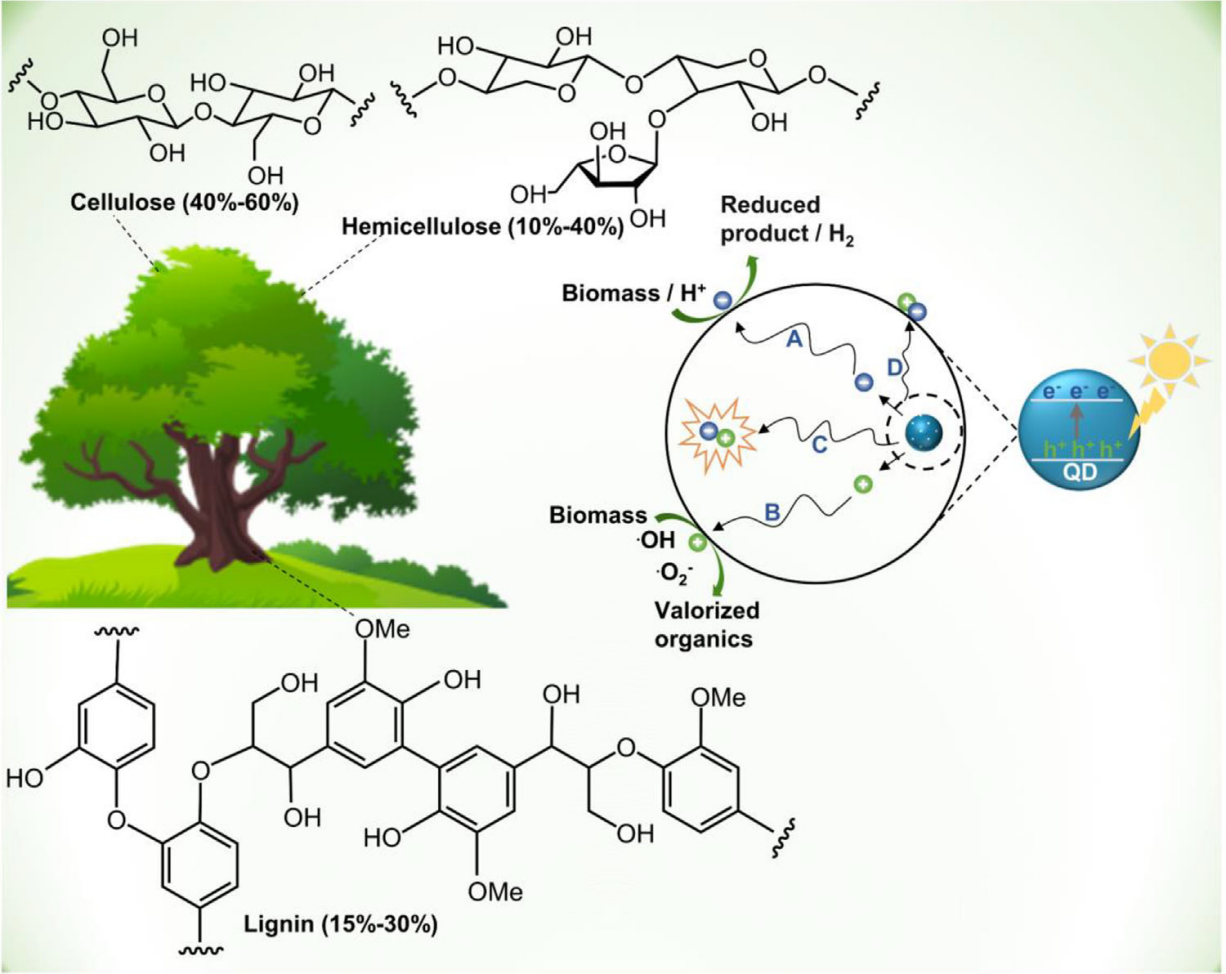
The diagram of chemical components of biomass and mechanism of QDs‐based photoreforming biomass.

Environmental issues and energy crises resulting from the massive depletion of fossil fuels are among the most extensively discussed and greatest challenges of the 21st century.^[^ ^1^, ^2^ ^]^ Therefore, the dual pressure of resources and the environment forces people to search for new renewable resources that can replace or partially replace the currently dominated fossil resources. As the largest renewable carbon resource on the planet, which is easily accessible, sustainable, and low cost, therefore making its efficient utilization attractive to reduce the conflict between the rising energy demand and deficient energy reserve.^[^ ^3^ ^]^ Depending on the sources of biomass, most plant raw materials are made up of lignocellulose biomass, which is also the most abundant, promising, and environmentally friendly source of green carbon that does not compete with food crops.^[^ ^4^, ^5^ ^]^ The three main polymers that make up the majority of lignocellulose's complex structure, including cellulose (40%–60%), hemicellulose (10%–40%), and lignin (15%–30%) combine in varying proportions hinging on the type of biomass (Figure 1, left).^[^ ^6^ ^]^ Hemicelluloses are branched polysaccharides that are surrounded by cellulose. Lignins are crosslinked phenolic polymers that are surrounded by two cellulosic polymers, which further form microfibrils that are resistant to chemical oxidation.^[^ ^7^ ^]^ Celluloses and hemicelluloses are saccharide macromolecules that are made of C5 or C5+C6 sugars that can be hydrolyzed to produce monosaccharides like glucose, arabinose, and xylose.^[^ ^8^ ^]^ Besides, lignin has the maximum important provenience of long‐term aromatics for the production of monocyclic aromatic compounds. Therefore, biomass and its derivatives are the only renewable resources that can provide a variety of organic carbons, thus presenting great potential for producing a variety of high‐value‐added chemicals and clean energy. It is estimated that the proportion of biomass‐derived chemicals and products will reach 35% by 2030.^[^ ^9^, ^10^ ^]^

Photocatalytic conversion of biomass (or biomass photoreforming) is usually performed reaction with ambient temperature and pressure, which shows great advances compared to traditional thermocataltyic strategies such as pyrolysis and gasification that usually require harsh conditions of high temperature, pressure, and use of explosive hydrogen.^[^ ^11^, ^12^ ^]^ In recent decades, substantial works have been dedicated to the study of various photocatalysts for photocatalytic biomass valorization.^[^ ^13^, ^14^ ^]^ Among the explored photocatalysts, QDs are quasi‐zero‐dimensional nanomaterials and a class of semiconductor nanoparticles consisting of a small number of atoms whose particle size is smaller than or close to the excitonic Bohr radius of the corresponding semiconductor material.^[^ ^15^ ^]^ Compare to other conventional semiconductor materials, QDs have emerged as one of the most promising candidates for biomass photoreforming (e.g., 5‐hydroxymethylfurfural (HMF),^[^ ^16^ ^]^ furfural,^[^ ^17^ ^]^ levulinic acid,^[^ ^18^ ^]^ and diol^[^ ^19^ ^]^) by reason that their unique properties, including broad range of light absorption, suitable band structure and adjustable, reproducibility and so on.^[^ ^20^ ^]^ In addition, the oxidation‐reduction potential of QDs can be tailored for a variety of photocatalytic systems by changing the framework and surface properties (size, shape, elemental composition). QDs are usually made up of groups II–VI, III–V, or IV elements. The diameter of typical QDs between 2 and 20 nm, which can increase numerous surface positive sites and that facilitate the adjustment of the bandgaps for sufficient light absorbance for either direct mineralization of organic materials or indirect oxidation of targeted compounds via •OH.^[^ ^21^ ^]^ The excited state of the QDs during solar irradiation drives the biomass photoreforming reaction (Figure 1, right). QDs photocatalyst was activated after absorbing light with exciton energy adequate or higher than their bandgaps. Then, the electron‐hole (e^−^‐h^+^) pairs are generated on the conduction band (CB) and valence band (VB) respectively, which can further transfer to the active locations of QDs to participate in the reaction. The e^−^ can reduce biomass to value‐added biochemicals or reduce proton (H^+^) to H_2_ depending on different catalytic substrates of photocatalytic systems (pathway A). For example, Michael et al. prepared CdSe QDs photocatalyst that enables the conversion of lignin model benzylic alcohols into high value‐added guaiacols and acetophenones via broken C─O bond and oxidation‐reduction reaction. Nevertheless, biomass or its derivatives as the reductive substrates to substitute oxygen evolution reaction (OER, Δ*E* ^0^ = 1.23 V) for h^+^ consumption in photocatalytic water splitting for the H_2_ production system. The h^+^ serves as an oxidant, which combines with H_2_O or ─OH to produce hydroxyl radical (•OH) and O_2_ to generate superoxide radical (•O_2_^−^), then oxidizes biomass‐derived reactants or intermediates into corresponding chemicals. (pathway B).^[^ ^22^ ^]^ For example, David et al. prepared CdS/CdO*_x_* QDs photocatalyst that could photoreforming lignocellulose into aldehydes and carboxylic acid through biomass oxidation, while supplying e^−^ to reduce hydrogen ions into H_2_.^[^ ^23^ ^]^ However, some of the photogenerated electrons and holes will inevitably recombination inside QDs during the migration process (pathway C) or on the surface before utilization (pathway D).^[^ ^24^ ^]^ Therefore, it is critical that raise the utilization efficiency of photogenerated e^−^ and h^+^ to promote the photocatalytic activity.

The existing reviews mainly focus on transition metal or plasmonic metal photocatalysts, while the comprehensive reviews centered on QDs‐catalyzed photocatalytic biomass valorization are still very limited. Herein, the review offers a timely summarization of the latest achievements in QDs‐catalyzed photocatalytic biomass valorization, with special emphasis on the catalysts or ligands design principles for efficient valorization. First, the designing principles of tuning the particle size, structure, and surface ligands are noted as the critical elements affecting the photocatalytic efficiency and selectivity of QDs. Then, the photocatalytic applications of QDs for various biomass transformations (e.g., lignocellulose, lignin, cellulose/hemicellulose, and their derivatives) are particularly discussed. Lastly, difficulties currently faced and development prospects are addressed for the practical application of QDs toward valorizing renewable biomass resources into high‐value chemicals and fuel for low‐carbon economy and development.

## 2 FACTORS AFFECTING QDS PHOTOCATALYTIC EFFICIENCY

Generally, strong light capture and exciton production, prolonged exciton lifetime, and quick surface reaction are the three necessary conditions for efficient photocatalytic applications. Therefore, precise modulation of the structure, electronic properties, and surface characteristics of photocatalysts is essential to improve the photocatalytic performance. A significant outcome of the quantum size effect in QDs photocatalysts is that the specific surface area boosts as grain diameter shrinks, which has a direct impact on the exciton properties, which improves the ability of e^−^–h^+^ to arrive at reactive sites, resulting in more effective redox reaction progress.

**FIGURE 2:**
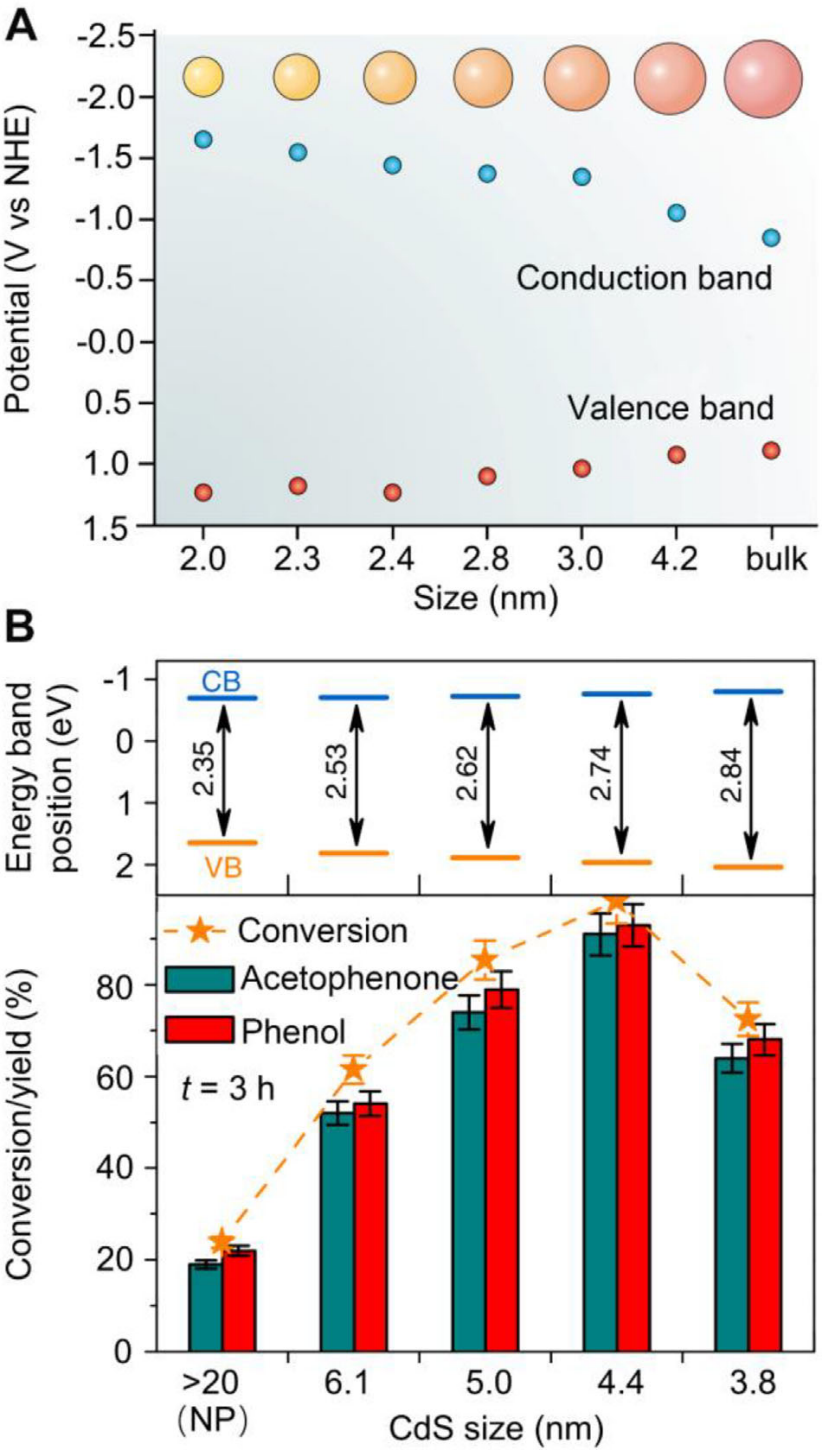
Schematic illustration of size change reflected in a corresponding bandgap and photocatalytic performance. A, Size‐dependent energy band positions of CdSe from the nanoscale to bulk. Reproduced with permission.^[^ ^28^ ^]^ Copyright 2018, Springer Nature. B, CB and VB of CdS in a range of size from 3.8 to >20 nm (top) and corresponding yield of conversion PP‐ol into acetophenone and phenol in 3 h of reaction. Reproduced with permission.^[^ ^30^ ^]^ Copyright 2018, Springer Nature.

Another considerable result of the quantum size effect is that the smaller size will result in an enhancement in the forbidden band gap's width.^[^ ^25^, ^26^, ^27^ ^]^ As a result, QD semiconductor materials show prominent size‐dependent energy band places. As an example, Figure 2A shows that a middle band spacing exists in bulk CdSe (*E*_g_ = 1.74 eV), whereas 2.0 nm CdSe QDs exhibit a significantly larger bandwidth of 2.88 eV.^[^ ^28^, ^29^ ^]^ As a result, the CB increases whereas the VB decreases, indicating the enhanced reducibility of the photo‐induced electrons on the CB and oxidability of the photo‐induced holes on the VB, respectively. In this regard, lesser QDs are anticipated to participate faster in the interfacial separation and transmission of electron‐hole pairs than the larger QDs, which increases the energy of excitons to a greater extent and thus enhances photocatalytic performance. Li et al. developed a series of CdS QDs and nanoparticles (NPs) with average dimension fluctuating between 3.8 to >20 nm. According to Figure 2B, the photocatalytic performance of transform 2‐phenoxy‐1‐phenylethanol (PP‐ol) into acetophenone and phenol of CdS QDs was significantly increased compare with CdS NPs during 3 h reaction. The optimal diameter of CdS QDs was identified as 4.4 nm, achieving 99% transformation of PP‐ol with high produces of acetophenone (91%) and phenol (93%). Further decreasing the size to 3.8 nm was found to be detrimental, which decreased both the conversion of PP‐ol (73%) and yields of the targeted products (acetophenone, 64%; phenol, 68%), which was most likely caused by weak ability to absorb visible light due to the risen of the bandgap.

**FIGURE 3:**
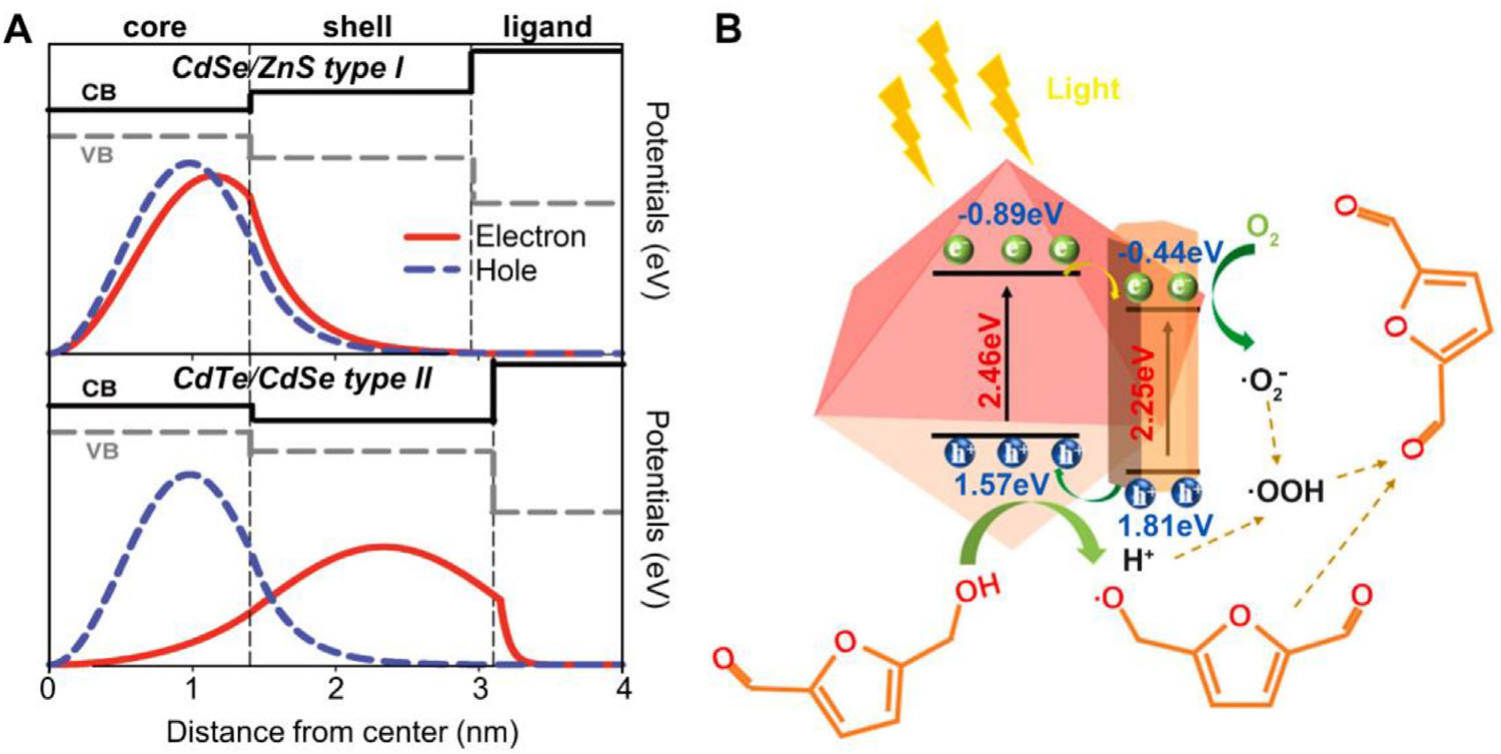
Diagram of core/shell structure radial distribution and transformation mechanism. A, Electron and hole radial direction curves of CdSe/ZnS (top half) and CdTe/CdSe (half bottom) core/shell QDs. Reproduced with permission.^[^ ^34^ ^]^ Copyright 2011, American Chemical Society. B, Diagram of heterojunction structure and HMF oxidation mechanism over Cd_1.5_In_2_S_4.5_. Reproduced with permission.^[^ ^35^ ^]^ Copyright 2022, Elsevier.

While the energy of electrons and holes can be increased by quantum confinement, it is considerable to consider that the excited state charges may occur quenching in the trap states during the transfer process. To improve the photocatalytic performance and stability, it is required to inhibit the competing channels for exciton recombination, including charge trapping and photocorrosion. Therefore, QDs‐based heterostructures for example core/shell structures, are usually adopted to boost photocatalytic effectiveness. The shell layer could help to decrease the outside flaw spot of the QDs and to lower the excitement complexation pathway. It is still unclear what determines how quickly charges move from the core to the reaction sites of QDs.^[^ ^31^ ^]^ The rapid e^−^–h^+^ separation and persistent lifetime of charge‐separated excited status (or moderative annihilation) are both indispensable for taking full advantage of charge carrier in QDs, and appropriate core‐shell spacing can contribute to the separation of e^−^–h^+^ and applied for photocatalysis.^[^ ^32^, ^33^ ^]^ Typically, the heterostructures based on QDs have two types including type‐I and type‐II, which depend on the band alignment of different components. For instance, Zhu et al. reported that designed two styles of core/shell structure‐based QDs, that is, CdSe/ZnS and CdTe/CdSe (Figure 3A).^[^ ^34^ ^]^ According to the energy band structure of type‐Ι QDs heterostructure, the core area is the main place where the electrons and holes existed because the CB and VB potential of the core are lower and higher than the shell, respectively.

The boundary of charge carrier appeared in type‐ΙΙ QDs heterostructure different from type‐Ι that electrons are mainly in the shell layer while holes are in the core layer, electrons and holes can be efficiently separated due to the confinement effect. The type‐ ΙΙ QDs heterostructure forms through the growth of shell materials with conduction and valence band energy levels that are more positive (or negative) than those in the core. As a result, the type‐ΙΙ QDs heterostructure was found to be a better photocatalyst because of its specific overspeed charge separation and migration properties, long unitary and numerous exciton lifetimes in comparison to type‐Ι. Zheng et al. synthesized Fe_3_O_4_@CdS@CQDs tripartite heterostructure via a hydrothermal process that CdS and carbon QDs formed step by step on the surface of Fe_3_O_4_.^[^ ^36^ ^]^ The in situ modified CQDs act as charge intermediaries to accelerate the photoinduced e^−^–h^+^ separation and provided reaction‐activated points. Benzyl alcohol is one of the typical biomass derivatives, which is selectively converted to benzaldehyde (57.22 mmol g_CdS_^−1^·h^−1^) accompanied by H_2_O_2_ production (27.06 mmol g_CdS_^−1^·h^−1^) with high production rates when using Fe_3_O_4_@CdS@CQDs as photocatalyst. In addition to the core/shell heterostructure, QDs can also form heterostructure structures with different crystalline phases or other traditional semiconductors. As an example, Zhang et al. reported the Cd*_x_* In*_y_* S_(_ *_x_*_+1.5_ *_y_*_)_ catalysts with a control mass of Cd:In proportions through the hydrothermal method.^[^ ^35^ ^]^ The outcome demonstrated that Cd:In ratio should be 1.5:2 with a maximum conversion of 5‐hydroxymethylfurfural (HMF) (68.8%), following selectivity 62.7% of 2,5‐diformylfuran (DFF), and 43.2% of DFF since reaction 6 h. The powerful heterojunction in Cd_1.5_In_2_S_4.5_ is made up of CdIn_2_S_4_ and CdS, which accurately aided in the transfer of charge carriers and radicals into reaction sites and enhanced the effectiveness of HMF photocatalytic conversion (Figure 3B).

**FIGURE 4:**
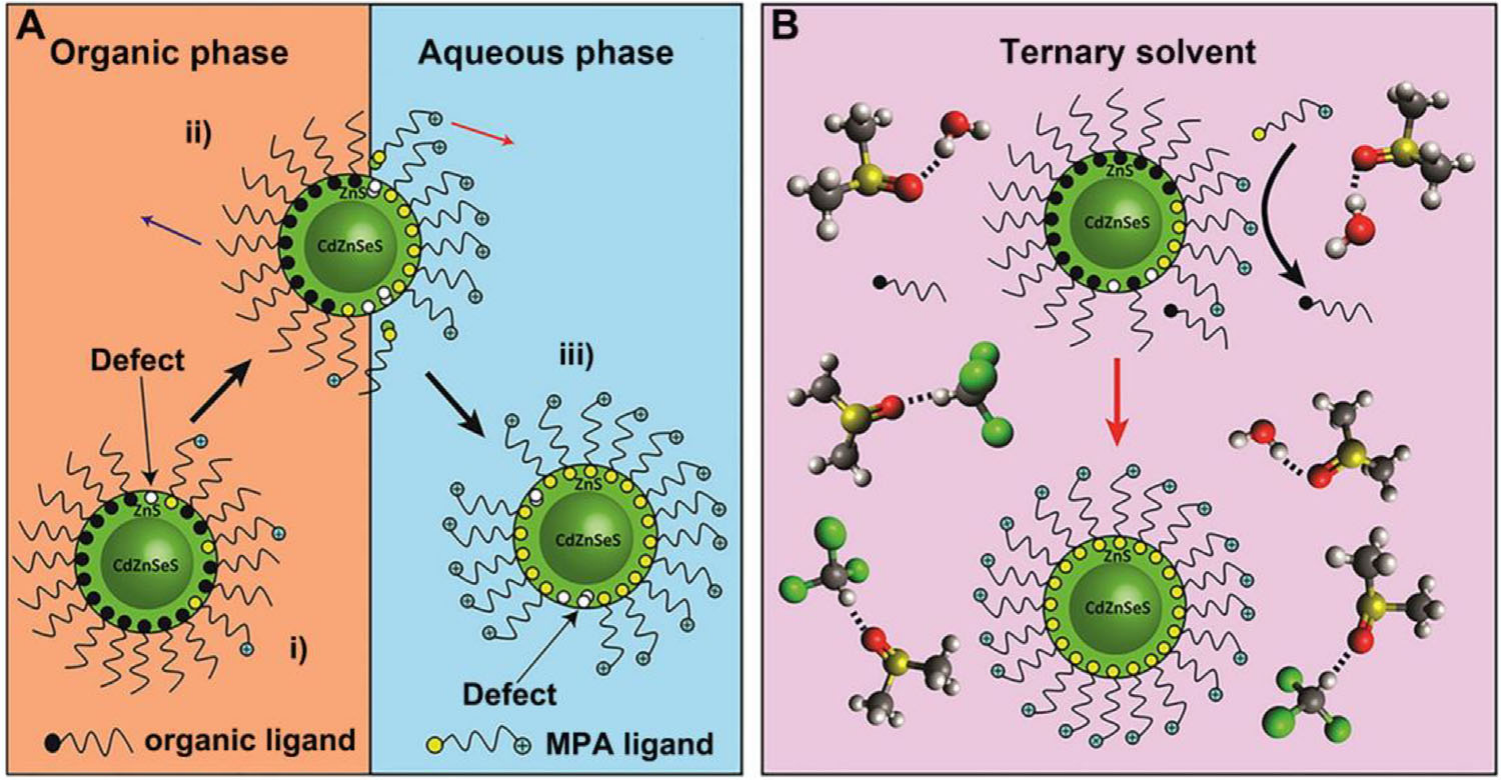
Schematic illustration of ligand exchange. A, Schematic of traditional biphase transfer. B, Ternary solvent systems of ligand exchange. Reproduced with permission.^[^ ^45^ ^]^ Copyright 2020, Elsevier.

In addition to the size and structure regulation, surface modification also is crucial to the synthesis, properties, processing, and applications of QDs. The multitudinous ligands of QDs significantly affect their facial characteristics, which can enhance catalytic activity by surface functionalization of QDs and controlling charge shift.^[^ ^37^, ^38^, ^39^ ^]^ The main roles of ligands are protecting QDs from aggregating, adjusting the characters of catalysts by changing chemical components the interface, and creating a secure pathway for charges carrier to migrate from catalysts to reaction substrates. Therefore, the catalysts with shorter ligands usually have better catalytic performance, due to the shorter electron transfer channels and quicker charge‐transfer capacity.^[^ ^40^ ^]^ Since QDs with uniform size and perfect lattice structure are usually synthesized in the organic phases, ligand exchange skills have been invented to resolve the immiscible problem of aqueous systems.^[^ ^41^, ^42^, ^43^ ^]^ However, it is unavoidable to generate a significant amount of surface defects during the process of ligand exchange process in the traditional biphase‐transfer system (Figure 4A). In this regard, Reinhart et al. found that the quantity of 3‐mercaptopropionic acid (3‐MPA) and stability have strong effects on the spectral absorption of the QD when performing exchange ligand with various apolar solutions.^[^ ^44^ ^]^ In another study, Le et al. built CdZnSeS/ZnS core/shell QDs with 3‐MPA modification from ligand exchange in a ternary solvent system composed of chloroform/water/dimethyl sulfoxide, which could effectively prevent physical perturbation by high interfacial tension between the organic phase and the aqueous phase thus have lower defect and fewer superficial radiationless recombination with high quantum yield (Figure 4B).^[^ ^45^ ^]^ They further proved that chloroform is a suitable organic solvent for ligand exchange in terms of solubility and suppressed quenching of QDs under ambient conditions. For instance, Wu et al. prepared a series of CdS QDs capped with different ligands by ligand exchange method, which dispersed in chloroform/methanol and used tetramethylammonium hydroxide to keep pH = 11. The formed CdS catalysts with different ligands (e.g., 1‐hexanethiol (HOL) (CdS‐HOL), oleic acid (OA) (CdS‐OA), and 6‐mercaptohexanoic acid (MHA) (CdS‐MHA)) were applied for photocatalytic conversion of birch woodmeal into aromatic monomers. They demonstrated that the ligands could mediate the charge transfer between QDs and reactants, and CdS with ligands have a better photocatalytic performance than ligand‐free CdS.^[^ ^46^ ^]^

The effects of organic ligands on the catalytic activity of QDs are controversial. In some cases, the organic ligands on the surface of heterogeneous catalysts have been viewed as harmful to catalytic activity, since the capping ligands often reduce the charge transfer efficiency and block the active locations for photocatalytic reactions.^[^ ^47^ ^]^ Hence, removal of the surface capping ligands is the key to ensuring satisfactory activity of semiconductor photocatalysts, such as CdS/TiO_2_,^[^ ^48^ ^]^ Au/TiO_2_,^[^ ^49^ ^]^ and CdS nanocrystals.^[^ ^50^ ^]^ In addition, some recent works have reported an increase in the performance of nanocatalysts in the presence of surface ligands.^[^ ^37^, ^46^, ^51^, ^52^ ^]^ A case in point is that Wu *et al.* constructed CdS QDs with organic ligands for photoreforming crude lignin into syringyl‐derived ketones (S‐ketones) and guaiacyl‐derived ketones (G‐ketones).^[^ ^46^ ^]^ The CdS QDs exhibited a lower conversion yield of products in the absence of ligands compare to that with an organic ligand (MHA). Since QDs have an extensive particular surface area, the ligands are attached to a large number of surface sites. Therefore, the catalytic efficiency and selectivity can be simply controlled by adjusting the properties of surface organic ligands.^[^ ^37^, ^46^ ^]^

## 3 APPLICATIONS OF QDS FOR PHOTOCATALYTIC BIOMASS VALORIZATION

Since the first report of using biomass as the photoreforming substrate in the 1980s,^[^ ^53^ ^]^ researchers have developed numerous QDs‐based photocatalysts and methods for biomass photoreforming including heterojunction and component regulation to manage the oxidation‐reduction properties of charge carriers.^[^ ^54^, ^55^, ^56^, ^57^ ^]^ QDs can be applied in various photocatalytic substrates conversion, such as lignocellulose, lignin and model compounds, cellulose/hemicellulose and their derivatives, and other biomass derivatives.^[^ ^27^, ^37^, ^58^, ^59^, ^60^ ^]^

### 3.1 Photocatalytic conversion of cellulose/hemicellulose and their derivatives

**FIGURE 5:**
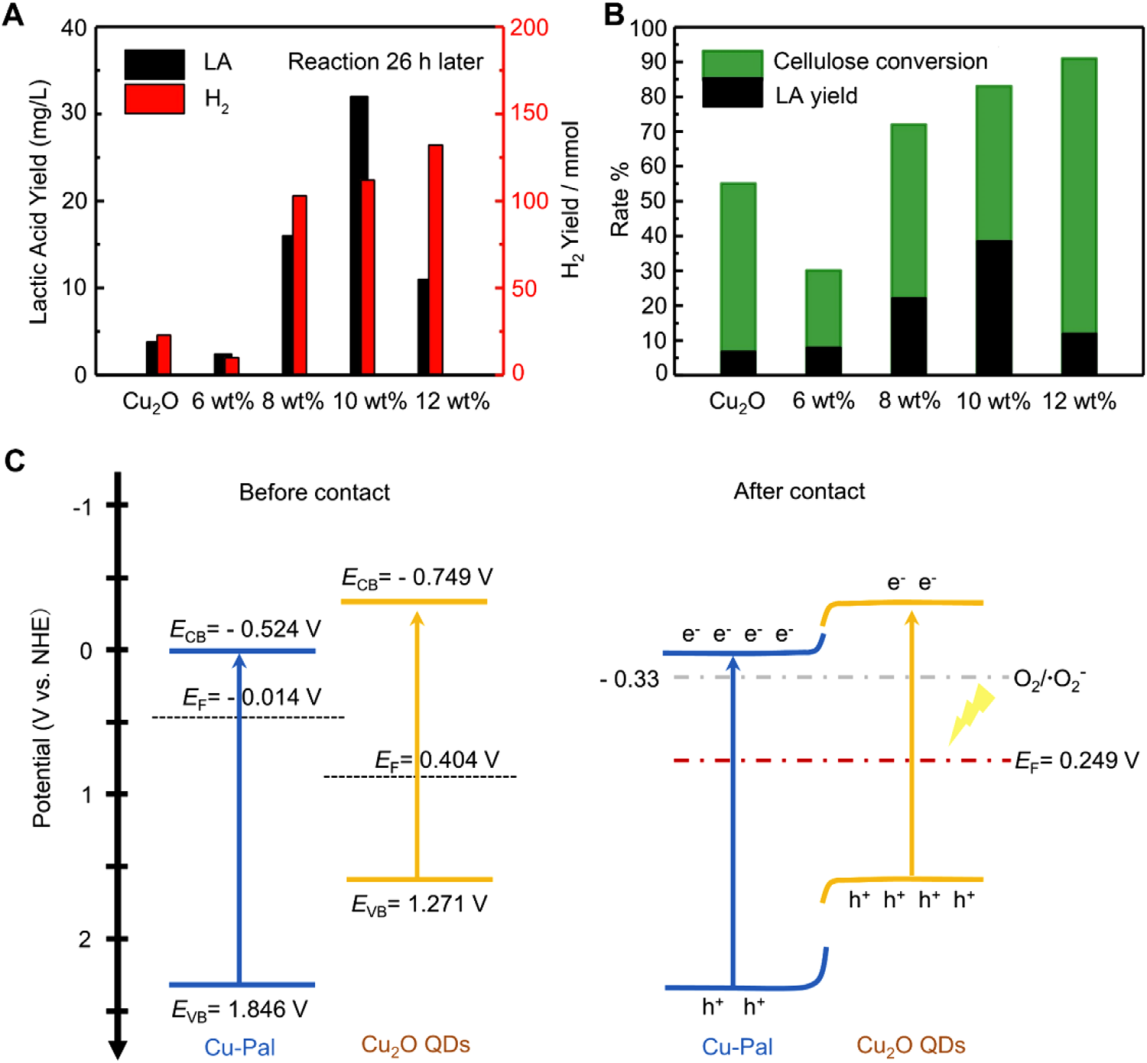
Photocatalytic performance of cellulose transform into LA and band structure of Cu_2_O QDs/Cu‐Pal heterostructure. A, The products conversion yield using Cu_2_O QDs/Cu‐Pal with various loading of Cu_2_O QDs. B, The graph of cellulose conversion rate and LA yield at various loading of Cu_2_O QDs. C, Band structure change of Cu_2_O QDs/Cu‐Pal before and after contact. Reproduced with permission.^[^ ^66^ ^]^ Copyright 2022, Elsevier.

The most prevalent component of biomass in nature is cellulose, a linear polymer of D‐glucose jointed by *β*−1,4 glycosidic bonds.^[^ ^61^, ^62^ ^]^ A lot of research has gone into creating catalytic processes that can transform cellulose/hemicellulose into chemicals with higher value, like polyhydric alcohol, organic acids, and furfuran‐derived compounds, which has received extensive attention.^[^ ^63^, ^64^, ^65^ ^]^ For example, Zhong et al. constructed the Cu‐doped natural palygorskite (Pal) catalyst, when the mass ratio of Cu was more than 6 wt%, the additional Cu_2_O QDs assembled with Cu‐Pal formed Cu_2_O/Cu‐Pal heterostructure.^[^ ^66^ ^]^ On account of Cu_2_O/Cu‐Pal heterostructure could efficiently encourage charge transfer while exposing numerous Lewis acid sites that allow for collaborative binding and transformation of intermediates, which exhibited remarkable photocatalytic performance and stability for converting cellulose into lactic acid (LA) maximum reached 32 mg L^−1^ at 10 wt% Cu_2_O QDs/Cu‐Pal and H_2_ maximum reached 132 mmol at 12 wt% Cu_2_O QDs/Cu‐Pal (Figure 5A). Then they found that an overabundance of Cu may have contributed to the agglomeration of Cu_2_O QDs, which then led to the oxidation of LA. The cellulose conversion rate increased from 30% to 91% with the loading of Cu_2_O QDs increased from 6 wt% to 12 wt%, while the yield of LA reduced at 12 wt% sample (Figure 5B). Furthermore, they proposed a reaction mechanism in which the CB and VB of Cu‐Pal wind up at the interface after forming a Cu_2_O/Cu‐Pal type‐II heterostructure, while the Fermi level of Cu_2_O QDs is lower than that of Cu‐Pal, causing the Cu_2_O QDs to bend downward (Figure 5C). It can promote the separation and migration of photoexcited carrier charges among Cu‐Pal and Cu_2_O QDs. As a result, the photocatalytic cleavage of the *β*−1,4‐glycosidic bonds and converting cellulose into glucose can be promoted.

The glucose unit of cellulose can be also used as the substrate for photoreforming. Zhao et al. reported that the CQDs/TiO_2_ photocatalyst featuring particular colored CQDs could improve the transformation of glucose coproduction of arabinose (a high‐value sugar) and gas fuels (H_2_ and CO) under neutral conditions.^[^ ^59^ ^]^ The certain colored CQDs from different carbon sources demonstrated numerous doping states, and external functional groups could enhance the capacity for charge separation of CQDs/TiO_2_ catalyst. The CQDs/TiO_2_ with dark green CQDs have optimal hydrogen production (up to 2.43 mmol h^−1^·g^−1^) and CQDs/TiO_2_ with red CQDs possess the highest arabinose production (up to 0.44 g L^−1^). Then they proposed the photocatalytic mechanism that CQDs could trap part of photogenerated e^−^ and then attract proton reduction to generate H_2_ then the remaining e^−^ interacts with consumed oxygen to create •O_2_^−^. In the meanwhile, h^+^ reacts with water to form •OH. The interaction of glucose with •O_2_^−^and •OH results in the formation of gluconic acid, which decomposition into arabinose and formic acid after the decarboxylation reaction. Then CO is produced through the formic acid dehydration process. Liu et al. constructed CIS@FSM composite material via anchoring CuInS_2_ QDs on defect‐rich graphene oxide (GO) for the photocatalytic xylulose creation of xylonic acid.^[^ ^67^ ^]^ They demonstrated that both defect density and crystallinity have a significant impact on charge transfer, and quantities of O‐containing compounds in GO that correlated with defect density were determined to be crucial for the adsorption of reactants, which can be adjusted by changing the thermal treatment temperature. However, the selectivity of xylonic acid was not sufficient due to the formation of other byproducts, such as formic acid and pyruvaldehyde. In another report, Yang et al. prepared CQDs@4CzIPN photocatalyst made up of carbon QDs integrated with 1,2,3,5‐tetrakis(carbazole‐9‐yl)−4,6‐dicyanobenzene (4CzIPN), which shown outstanding universality for photocatalytic converting biomass‐derived monosaccharides into lactic acid with a maximum yield of 96.9%.^[^ ^68^ ^]^ In addition, cellulose can also be combined with QDs for photocatalytic degradation of dyes, drug delivery, and antimicrobial applications.^[^ ^69^, ^70^, ^71^ ^]^

**FIGURE 6:**
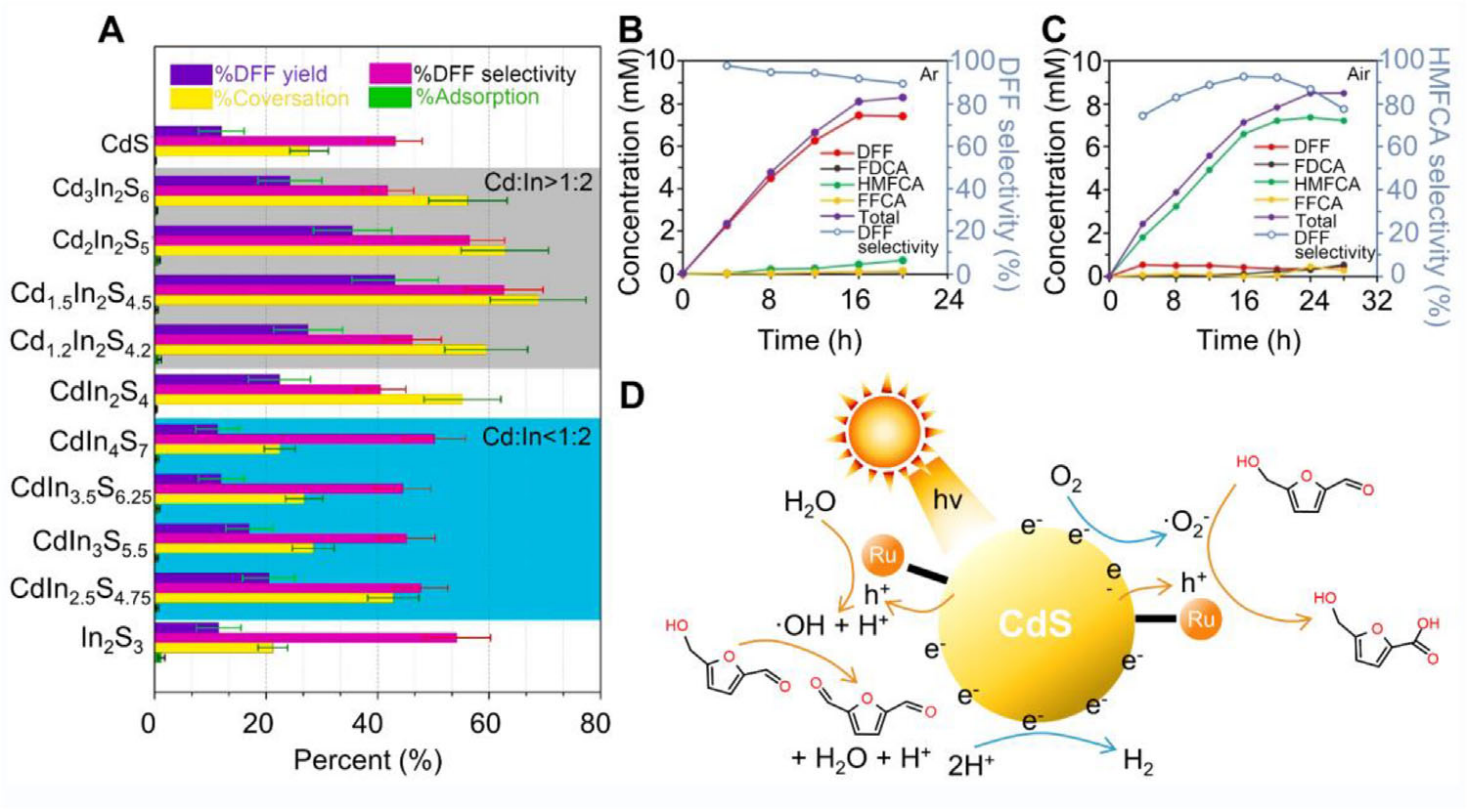
Photocatalytic conversion and mechanism of HMF over QDs‐based catalyst. A, Results of the oxidation of HMF and the adsorption rate of CdS, CdIn_2_S4, In_2_S_3,_ and Cd*_x_* In*_y_* S_(_ *_x_*_+1.5_ *_y_*_)_ composites. Reproduced with permission.^[^ ^35^ ^]^ Copyright 2022, Elsevier. B, The HMF concentration over time and DFF selectivity using Ru‐CdS catalyst under Ar ambience. C, The concentration over time and HMFCA selectivity using Ru‐CdS catalyst under air atmosphere. D, Schematic mechanism of Ru‐CdS photocatalytic conversion HMF. Reproduced with permission.^[^ ^77^ ^]^ Copyright 2022, Wiley‐VCH GmbH.

HMF and furfural are the key intermediates in cellulosic biomass conversion, which are significant industrial and pharmaceutical chemicals that originate from the depolymerization of C6 carbohydrates.^[^ ^72^, ^73^ ^]^ The United States Department of Energy has listed HMF as value‐added biomass‐derived platform chemicals and has garnered significant global research attention in recent years.^[^ ^74^, ^75^, ^76^ ^]^ In the biochemical and medicinal fields, 2,5‐diformylfuran (DFF), 5‐hydroxymethyl‐2‐furancarboxylic acid (HMFCA), 5‐formyl‐2‐furancarboxylic acid (FFCA), and 2,5‐furandicarboxylic acid (FDCA), HMFCA, 5‐FFCA, and FDCA are essential chemical raw materials, which can be produced by oxidizing HMF with two aldehydes (─CHO) and hydroxyl (─OH) functional groups. However, targeting precise oxide HMF to produce specific products is extremely challenging because the ─CHO and ─OH functional groups are prone to oxidation and metamorphism. Zhang et al. prepared Cd*_x_* In*_y_* S_(_ *_x_*_+1.5_ *_y_*_)_ photocatalysts with a unique ratio of Cd:In for the photocatalytic transformation of HMF into DFF in water (Figure 6A).^[^ ^35^ ^]^ The optimal Cd:In ratio was determined to be 1.5:2, with 68.8% conversion of HMF, 62.7% DFF selectivity, and 43.2% DFF yield. An effective heterojunction is formed inside the Cd_1.5_In_2_S_4.5_, which promotes the transfer of long‐distance charge carriers and results in greatly optimized photocatalytic performance in HMF conversion. with long‐distance charge separation and promote the transfer of photogenerated charge carriers, resulting in greatly improved photocatalytic performance in HMF conversion. It was further testified that O_2_, •O_2_^−^, electrons and holes are identified as the active sites for selectively oxidizing HMF to DFF.

Xia et al. created Ru‐CdS photocatalyst through Ru complex combined with CdS QDs for selective catalytic oxidation of HMF into DFF or HMFCA by control reaction atmosphere conditions.^[^ ^77^ ^]^ Note that this was the primary study reporting the selective production of HMFCA via photocatalytic HMF oxidation. Since Ru complexes enable efficient extraction and fast transfer the holes from CdS QDs to HMF, DFF could be obtained in high selectivity of 91.8% under an Ar atmosphere (Figure 6B). In contrast, HMFCA was manufactured with a selectivity of 92.6% in an air atmosphere after 17.5 h of reaction (Figure 6C). They further demonstrated the selectivity of DFF and HMFCA are related to •OH (Ar atmosphere) and •O_2_^−^ (Air atmosphere) radicals by adding tert‐butanol (TBO) and 5,5‐dimethyl‐1‐pyrroline‐*N*‐oxide (DMPO) free radical trapping agent, which resulted in significantly reduced conversion of HMF to DFF and HMFCA, respectively. Finally, a tentative mechanism was proposed (Figure 6D). The generated h^+^ first move from CdS QDs to Ru complexes for oxidizing water formed •OH reaction with HMF to produce DFF under Ar atmosphere, while photogenerated e^−^ and O_2_ reaction formed •O_2_^−^ for selective oxidation of HMF into HMFCA under air atmosphere. Besides, Yuan et al. engineered a new CdS QDs/*x*Ch composite photocatalyst through CdS QDs load on the chitosan carbon with N‐doped (Ch) by hydrothermal method, which achieved prominent photocatalytic performance with HMF conversion of 78.73% and DFF selectivity of 97.31% at 20% Ch loading (CC‐20).^[^ ^78^ ^]^

### 3.2 Photocatalytic conversion of lignin and its model compounds

**FIGURE 7:**
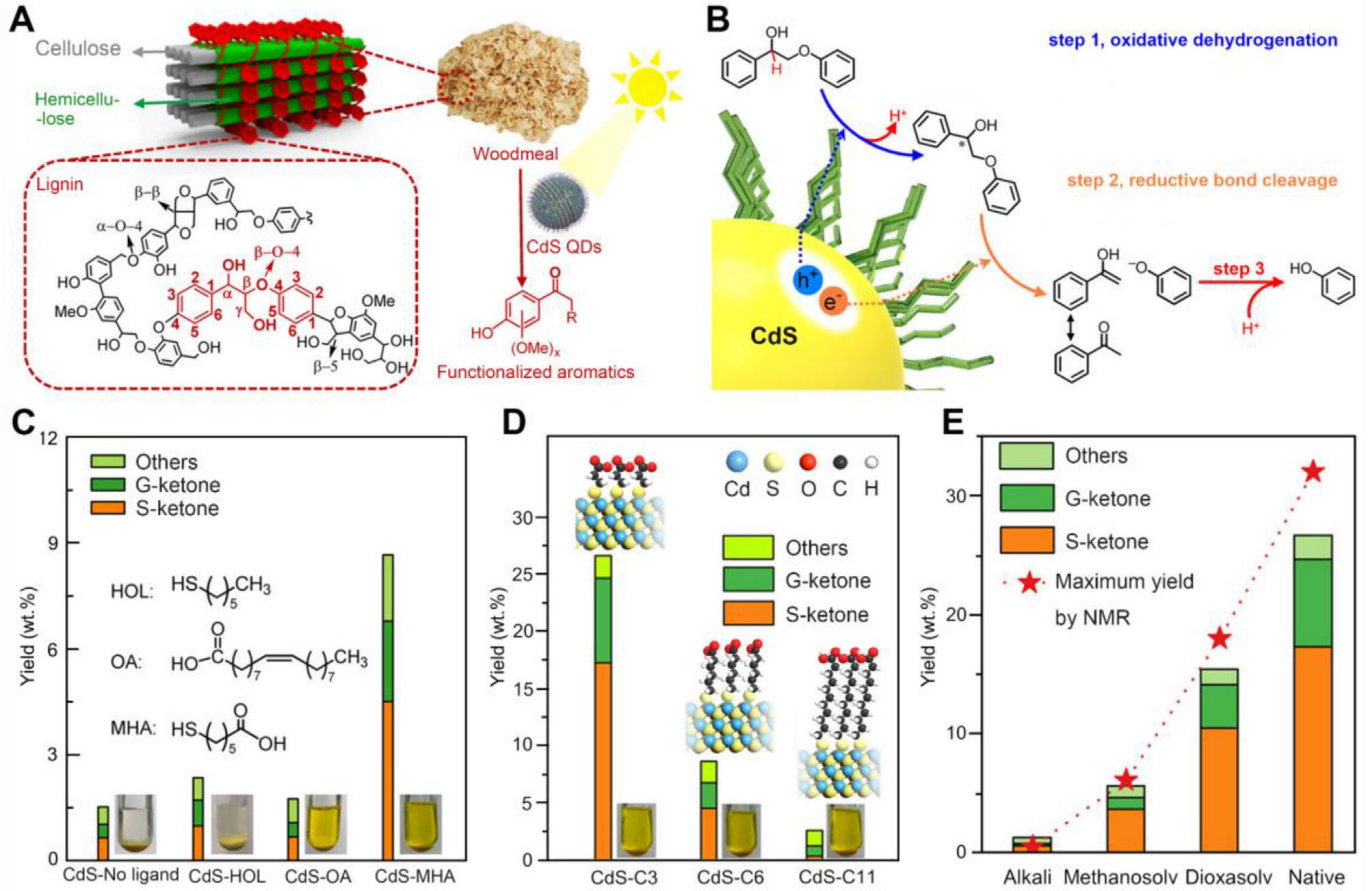
Photocatalytic conversion yield and mechanism of nature or model compound lignin over QDs‐based catalyst with different ligands. A, Schematic structure of lignin and CdS QDs photocatalytic conversion of woodmeal. B, Diagrammatic representation of the conversion of PP‐ol by ligand‐mediated electron‐hole coupling. C, Product yield diagram of photocatalytic birch powder on CdS QDs with various ligands. D, Product yield graph of photocatalytic birch powder on CdS QDs with different length of ligands. E, Yield graphic of aromatic monomers through photocatalytic conversion lignin from diverse sources. Reproduced with permission.^[^ ^37^ ^]^ Copyright 2019, American Chemical Society.

Lignin is the most rigid content among the three constituents of lignocellulosic biomass, which is produced through the enzyme‐mediated dehydrogenation of aromatic alcohol precursors made up of l‐phenylalanine building blocks. It is in charge of transporting water and nutrition, offering stability and support, and shielding the plant from diseases and environmental challenges. The main building blocks of lignin are three phenylpropanol precursors: sinapyl, coniferyl, and p‐coumaryl alcohols. The aromatic units are randomly intertwined into complex reticulated polymers structure of lignin by a series of carbon‐carbon and ether linkages, thus hindering the effective transformation of lignin into highly‐valued aromatics (Figure 7A).^[^ ^37^, ^79^ ^]^ The main structure of the lignin macromolecule contains a large number of *β*‐O‐4 connections, which make up 45–60% of all linkages and have a bond dissociation energy (BDE) of 56.54–72.30 kcal mol^−1^.^[^ ^80^ ^]^ Therefore, breaking *β*‐O‐4 linkages in lignin is the most crucial and effective method for valorizing lignin into fine chemicals, polymers, and fuels.^[^ ^81^, ^82^ ^]^ For instance, Wu et al. designed CdS QDs can selective broken *β*‐O‐4 bonds of native lignin effectively when illuminated by visible light (Figure 7A), through a three‐step electron‐hole coupled (EHCO) photoredox route (Figure 7B).^[^ ^37^ ^]^ Then, they systematically investigated the impact of various surface ligands on photocatalytic transformation native lignin into functionalized aromatics, which main contains S‐ketones and G‐ketones. Specifically, the CdS QDs covered a hydrophilic ligand of MHA, were pseudo‐homogeneous in polar CH_3_OH/H_2_O solution, which permitted close contact between QDs and the reactant, enhancing the conversion yield of lignin. Instead, the CdS QDs with a ligand of 1‐hex‐anethiol (HOL) readily aggregated in solvents and thus only gained an inferior yield (2.4 wt%) than CdS‐MHA (8.7 wt%) of aromatic monomers (Figure 7C). However, they found that CdS‐OA QDs modified with oleic acid (OA) ligand was dissolved adequately in hexane solvent, but the product yield was only 1.8 wt%. (Figure 7C). Thus, they speculated that the ligand ought to perform else functions besides encouraging the formation of colloidal solutions to improve the photocatalytic activity.^[^ ^83^ ^]^ Then, they demonstrated that the length of the alkyl chain in ligands impacts on photocatalytic performance of the catalyst in native lignin conversions through CdS QDs with various ligands including 3‐MPA (C*_n_* = 2, *n* represents the number of alkyls), MHA (C*_n_* = 5), and 11‐mercaptoundecanoic acid (MUA C*_n_* = 10). Among these catalysts, the CdS‐C3 QDs showed a satisfactory product yield conversion rate of 27 wt% in comparison with CdS‐C6 (9 wt%) and CdS‐C11 (3 wt%) (Figure 7D). According to the photoelectrochemical study lignin model compound 2‐phenoxy‐1‐phenylethanol (PP‐ol), the Cn in the CdS‐Cn QDs controlled the speed of charge transfer between QDs and substrates, and a shorter length could result in a faster migration rate. Thus, they summarized a three‐step EHCO photoredox mechanism example‐based PP‐ol conversion into acetophenone and phenol (Figure 7B). The ligand afforded photogenerated e^−^/h^+^ transfer pathway from QDs to PP‐ol, in which h^+^ cleaved the C_α_‐H bond into a C_α_ radical and a proton in step 1. In the meanwhile, the BDE of *β*‐O‐4 bond in the Cα was reduced to 7.8 kcal/mol so that easily transform into phenoxy anion and acetophenone when the C*_α_* radical accepted the e^−^ from the CdS core in step 2. In step 3, the phenoxy negative ion oxidized to phenol by proton. In addition, this study explored the efficient catalytic conversion of lignins from various sources substrates into monomeric aromatics with near technical lignins yield using CdS‐C3 QDs system certification, which demonstrates its universality (Figure 7E). This work provided a new and useful approach to ligand modification for designing efficient QDs for native lignin biomass valorization.

**FIGURE 8:**
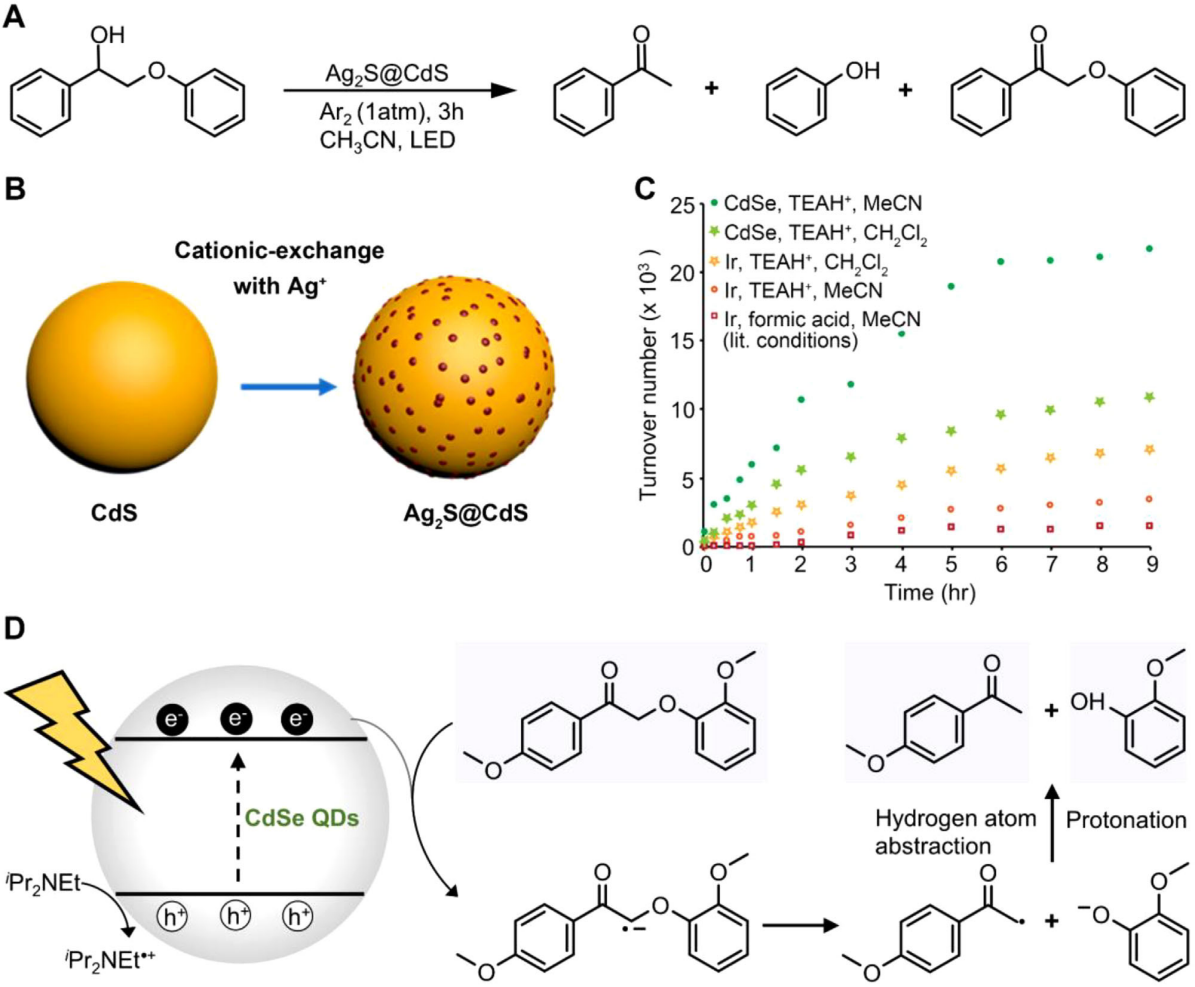
QDs photocatalytic cleavage *β*‐O‐4 bonds in the typical lignin model compounds. A, The schematic diagram of photocatalytic transformation PP‐ol using Ag_2_S@CdS. B, Molecular diagram depicting the Ag^+^ exchange process. Reproduced with permission.^[^ ^87^ ^]^ Copyright 2020, American Chemical Society. C, Turnover number conversion of the lignin model substrate 2‐(2‐methoxyphenoxy)−1‐(4‐methoxyphenyl) ethenone (BK) over the first 9 h of the reaction using CdSe QDs and iridium catalyst. D, Mechanism of PP‐ol photoreduction using CdSe QDs. Reproduced with permission.^[^ ^58^ ^]^ Copyright 2019, American Chemical Society.

On behalf of exploring the reaction mechanism, the majority of research efforts have chosen straightforward model compounds that are easily accessible and have typical chemical bonds found in natural lignin polymers.^[^ ^81^, ^84^ ^]^ 2‐(2‐methoxyphenoxy)−1‐(4‐methoxyphenyl) ethanol (BA) and PP‐ol commonly serve as the lignin model compounds, which could be easily analyzed and have simple structures.^[^ ^58^, ^85^, ^86^ ^]^ For example, Wang et al. reported that CdS NPs exhibited a distinctive ability in breaking the *β*‐O‐4 linkage of PP‐ol in comparison to TiO_2_, Cu_2_O, BiVO_4_, ZnS, CuS, and graphitic C_3_N_4_.^[^ ^30^ ^]^ Subsequently, Hyeonji et al. reported that Ag_2_S@CdS enabled significantly enhanced conversion (nearly 100%) of five typical lignin model compounds with high selectivity towards cleaved aromatic compounds (Figure 8A).^[^ ^87^ ^]^ Furthermore, a heterogeneous Ag_2_S@CdS structure was developed on CdS nanoparticles by Ag^+^ exchange method (Figure 8B). The Fermi level of CdS dramatically shifted with the introduction of Ag^+^ domains, which effectively promote the cleavage of *β*‐O‐4 bonds. In a parallel study, Wang and coworkers demonstrate that the yield of PP‐ol conversion of CdS QDs was superior to that CdS NPs and enabled selectively cleave *β*‐O‐4 bonds while additional surface oxygenic functional groups r almost remained unaltered.

Surprisingly, Michael et al. demonstrated that multifunctional CdSe QDs could selectively cleave C*_α_*‐O linkages in lignin model compounds.^[^ ^58^ ^]^ Furthermore, PP‐ol could be directly transformed into monomers without the need for any additional postprocessing process by single‐vessel conversion of CdSe QDs. The CdSe QDs were considered viable photoredox catalysts due to their high molar extinction coefficient, large surface area and prolonged excited states. As a result, the trans‐4‐cyanocinamate‐functionalized CdSe QDs exhibited excellent performance with a turnover frequency of 22,000, which was 15‐fold greater compared to the state‐of‐the‐art Ir catalyst (7000) (Figure 8C). Based on the findings, a tentative mechanism was proposed that CdSe QDs were effective for photocatalytic reductive cleavage of the *β*‐O‐4 connection with iPr_2_NEt as a hole quencher (Figure 8D). The lignin model substrates benzylic alcohol (BA) was oxidized to benzylic ketone intermediate by triethylammonium‐PF_6_ ([HNEt_3_]PF_6_), which was further photochemically reduced to high‐value acetophenones and guaiacols by CdSe QDs through phenyl ketone intermediates. However, the authors had not conducted additional catalytic tests on other native and lignin compounds such as woodmeal, paper, benzyl alcohol, and benzyl aryl ethers to verify the generalizability of the developed strategy. Besides, the toxic Cd element of CdS and CdSe QDs limited the integration into current biorefineries and industrial production.

### 3.3 Photocatalytic conversion of lignocellulose

Lignocellulose, a plentiful biomass on Earth with high functionality, possesses enormous potential for the sustainable production of functionalized chemicals. Given that these highly functionalized lignocellulose‐based molecules can travel along a wide range of paths to various products, their selective transformation is an essential target but remains a tough problem. Moreover, Conventional methods for converting lignocellulose, such as thermolysis and hydrogenolysis, typically need harsh operating cases and have low product selectivity.^[^ ^88^ ^]^ A promising method for lignocellulose transformations that can be carried out under benign environmental conditions is photocatalysis.^[^ ^89^, ^90^ ^]^

**FIGURE 9:**
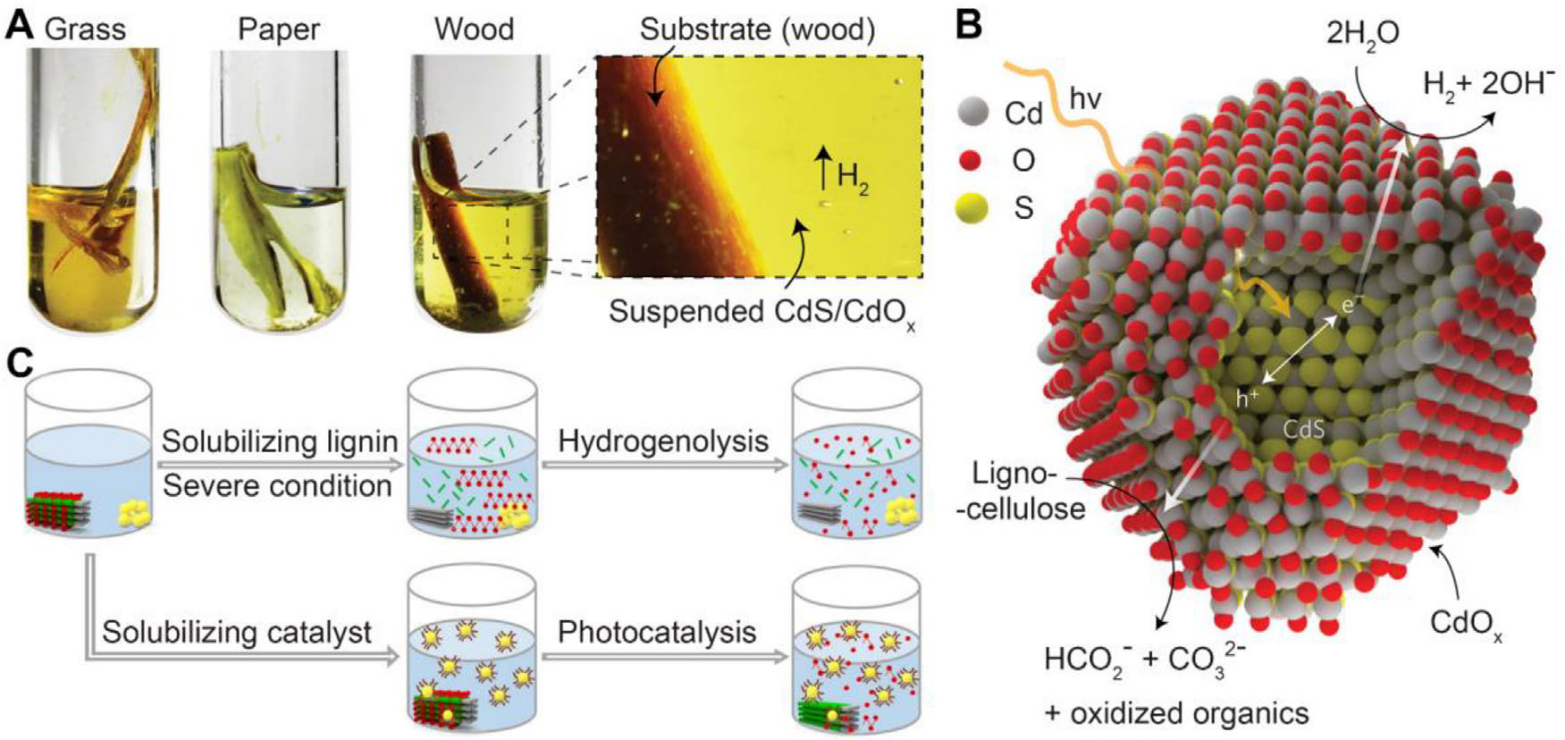
Photoreforming of lignocellulose to H_2_ over CdS/CdO_x_ and solubilizing catalyst method. A, Diagram of photocatalytic H_2_ production from raw biomass materials (grass, paper, and wood) in alkaline solutions. B, Schematic diagram of CdS/CdO*_x_* for lignocellulose photoreforming. Reproduced with permission.^[^ ^23^ ^]^ Copyright 2017, Springer Nature. C, Graphical representation of the solubilizing catalyst approach using CdS QDs in comparison to the conventional high‐temperature hydrogenolysis method for solubilizing lignin. In the mockups, the blocks composed of red, green and grey stand for lignocellulose; the yellow spheres stand for CdS QDs; the brown line stands for the ligand of CdS QDs. Reproduced with permission.^[^ ^30^ ^]^ Copyright 2018, Springer Nature.

However, photoreforming lignocellulose directly or extracted cellulose poses a significant challenge, due to expensive and inefficient lignocellulose refining.^[^ ^91^ ^]^ David et al. designed a photocatalytic system, which can reform unprocessed lignocellulose (e.g., wood, paper) to H_2_ under solar irradiation at room temperature using CdS/CdO*_x_* photocatalyst in a highly alkaline environment, presenting an inexpensive route to upgrade waste biomass in alkaline aqueous solution (Figure 9A).^[^ ^23^ ^]^ The formation of a surface layer of CdO*_x_* on CdS/CdO*_x_* can prevent the photocorrosion of CdS by blocking the defect sites on CdS that facilitate nonradiative charge carrier reorganization.^[^ ^92^ ^]^ Furthermore, they proposed an initial theory for how lignocellulose reforming on CdS/CdO*_x_*, that formed similar Cd‐O‐R bonds in lignocellulosic substrates bind to the CdS/CdO*_x_* surface, enabling the photocatalyst to convert alcohols to aldehydes quickly. Then, the aldehyde undergoes oxidation to yield a carboxylic acid, which is decarboxylated to CO_3_^2−^ while H_2_O is reduced to H_2_ as shown in Figure 9B. Surprisingly, Wu et al. proposed a lignin‐first concept that the lignin in biomass is catalyzed by valorization in the first step, realizing the utilization of the entire lignocellulosic biomass in an effective way.^[^ ^30^ ^]^ In this study, CdS QDs were identified as specific tools for accurate cleavage of *β*‐O‐4 bonds of lignin into functionalized aromatics efficiently, while cellulose/hemicellulose remains nearly intact. Differing from David's work that applied alkaline conditions to dissolve lignocellulose and other saccharides, this work presented a creative approach that applied soluble catalyst instead of lignin solubilization, which not only solves an inferior efficacy of solid‐solid interaction between the catalyst and substrate, but also represents a more energy‐efficient strategy than high‐temperature hydrolysis (Figure 9C). What is more, the colloidal CdS QDs can be detached from the reaction solution through an aggregation‐colonization strategy, as the colloidal nature of QDs can be controlled by highly dispersing or dissolving the organic ligand in an appropriate solvent. The lignin‐first approach makes it possible to maximize the use of lignocellulosic materials in temperate conditions.

### 3.4 Photocatalytic conversion of other biomass derivatives

**FIGURE 10:**
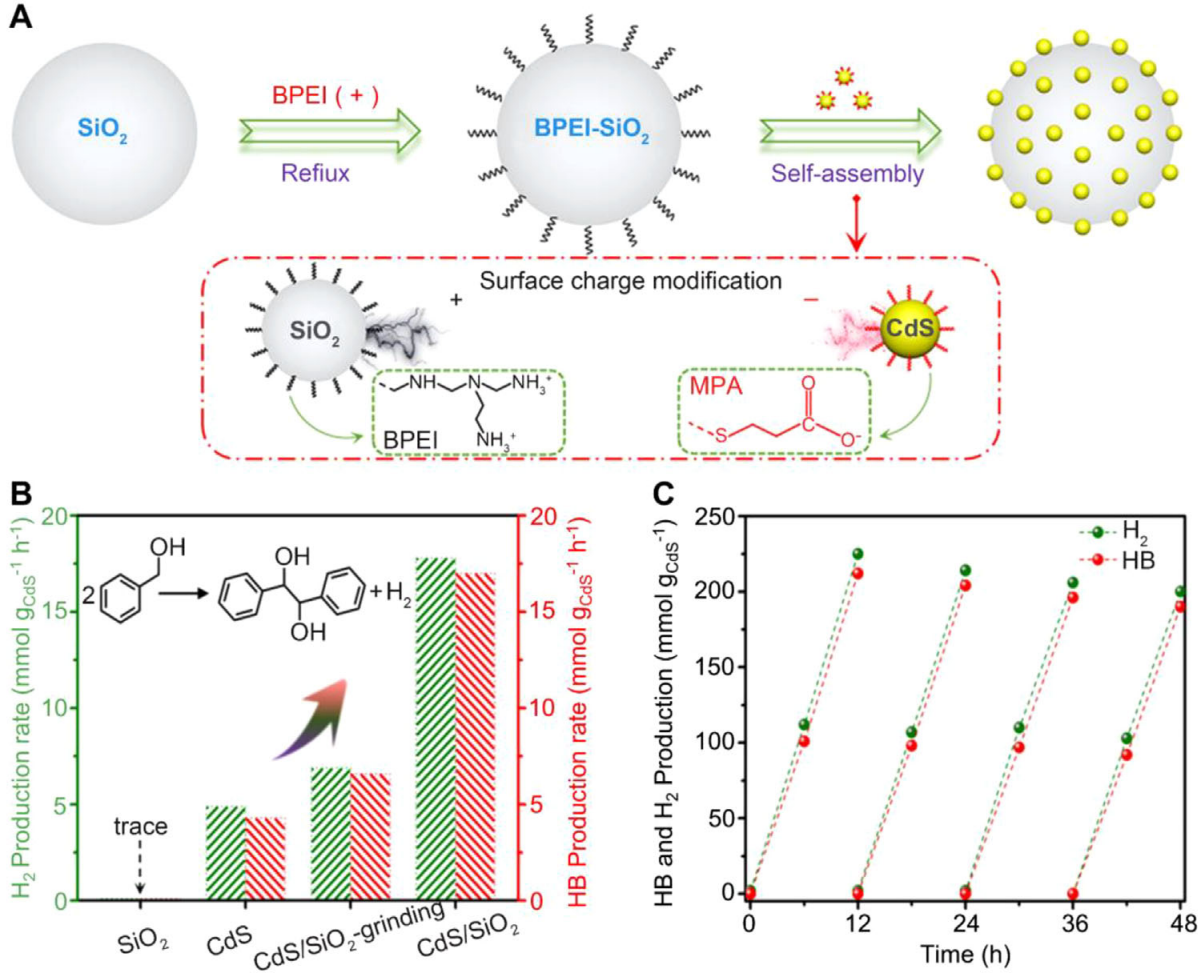
Schematic diagram of CdS/SiO_2_ composites synthesis route and photocatalytic conversion performance for transform BA into HB with the coproduction of H_2_. A, The synthesis procedure of CdS/SiO_2_ composites. B, The photocatalytic production rates of HB and H_2_ upon different catalysts. C, Recycling tests on 2.5% CdS/SiO_2_ composite. Reproduced with permission.^[^ ^55^ ^]^ Copyright 2020, American Chemical Society.

Other biomass derivatives (e.g., alcohols, amines, and sugars) have also been employed as the sacrificial reagents to consume holes for their selective oxidation to value‐added chemicals, while simultaneously coproduction of affordable H_2_ from proton reduction. Benzoyl alcohol (BA) is a significant platform chemical that can be produced from a range of biomass resources, which is also known as substituted aromatic alcohol. The hydrobenzoin (HB) and benzoin (BZ) are created from dehydrogenized BA, which can be used as flexible configurational substrates in fine chemicals and pharmaceutical intermediates with a broad variety of chiral and artificial chemistry applications.^[^ ^93^ ^]^ Qi et al. prepared SiO_2_‐supported CdS QDs (CdS/SiO_2_), which exhibited interesting performance that photoredox‐driven dehydrogenation behavior for efficient C─C coupling of BA into HB with the coproduction of H_2_.^[^ ^55^ ^]^ Due to existing covering ligands on CdS QDs and the ─COOH and ─NH_2_ groups on SiO_2_, CdS QDs were tight linked with SiO_2_ support via a static self‐assembly method (Figure 10A). CdS QDs could absorb the scattered light in the near field of the SiO_2_ support, resulting in significantly improved light‐harvesting capability and more efficient charge carrier generation while remaining the size unchanged. The CdS/SiO_2_ photocatalyst exhibited 3.7 times higher H_2_ production rate than individual CdS, which also achieved a high HB production rate of 17.0 mmol g_CdS_^−1^·h^−1^ (yield, 84.6%; selectivity, >99%) rather steady during four reaction cycles (48 h in total). Algae have the greatest potential among renewable biomass energy sources because of their senior process of photosynthesis activity and biomass harvest, as well as the lack of need for arable land for cultivation.^[^ ^94^ ^]^ In general, the high carboxylic acid content of bio‐crude oils has a negative impact on their quality. Therefore, carboxylic acids are usually changed to other harmless ingredients such as esters, to improve the quality of algal bio‐crude oil. Recently, Wang et al. employed CdS QDs photocatalyst for converting carboxylic acids into esters to improve the quality of oil by aggrandizing the ester percentage composition from 17.2% to 47.9%.^[^ ^27^ ^]^ CdS‐QDs also showed satisfactory recyclability with four cycles (12 h/cycle) using methanol as a solvent. The photogenerated e^−^ and h^+^ are formed by photocatalyst after being excited by an Xe lamp, which can react with methanol and algal biocrude oil respectively.^[^ ^95^ ^]^ The photogenerated h^+^ could oxidize carboxylic acid generating acyl free radicals on the VB. Meanwhile, CH_3_O• produced by methanol accepted e^−^ on the CB, then reaction with acyl free radicals transformed into esters. Finally, the rest of OH^−^ combined with H^+^ to form water (Figure 10B). As follows in Figure 10C, the activities of H_2_ and HB generation were rather steady during four reaction cycles (48 h in total).

Algae have the greatest potential among renewable biomass energy sources because of their senior process of photosynthesis activity and biomass harvest, as well as the lack of need for arable land for cultivation.^[^ ^94^ ^]^ In general, the high carboxylic acid content of bio‐crude oils has a negative impact on their quality. Therefore, carboxylic acids are usually changed to other harmless ingredients such as esters, to improve the quality of algal bio‐crude oil. Recently, Wang et al. employed CdS QDs photocatalyst for converting carboxylic acids into esters to improve the quality of oil by aggrandizing the ester percentage composition from 17.2% to 47.9%.^[^ ^27^ ^]^ CdS‐QDs also showed satisfactory recyclability with four cycles (12 h/cycle) using methanol as a solvent. The photogenerated e^−^ and h^+^ are formed by photocatalyst after being excited by a Xe lamp, which can react with methanol and algal biocrude oil respectively.^[^ ^95^ ^]^ The photogenerated h^+^ could oxidize carboxylic acid generating acyl free radicals on the VB. Meanwhile, CH_3_O• produced by methanol accepted e^−^ on the CB, then reaction with acyl free radicals transformed into esters. Finally, the rest of OH^−^ combined with H^+^ to form water.

## 4 SUMMARY AND PERSPECTIVES

Converting biomass into fuels and value‐added chemicals is imperative for the pursuit of sustainability in the future. Photocatalysis has developed into a promising method for converting biomass and its derivatives, allowing permits for targeted transformations of particular functional groups or bonds in the backbones as well as low cost and environmental friendly.^[^ ^84^, ^96^ ^]^ The development of photocatalyst equipment with high activity and selectivity is essential to strengthen the photocatalytic transformation performance of biomass. QDs stand out from the crowd of semiconductors photocatalysts that play a vital role in a variety of crucial transformations of biomass valorization. QDs have the advantages of the adjustable band gap, controllable size and ligand, selective cleavage of specific chemical bonds and easy preparation, which makes them a type of more ideal material for biomass valorization. Furthermore, there is a scarcity of reviews based on QDs photocatalysts for the transformation of biomass. Here, we briefly discussed the recent status of photocatalytic biomass valorization over semiconductor QD‐based materials. Then, the factors affecting QDs photocatalytic efficiency and regulation strategies of QDs were summarized, including size control, ligand regulation, and heterojunction construction. Afterward, the recent advances in QDs and their ligand regulation application in the photocatalytic conversion of biomass were discussed, including lignocellulose, lignin, and cellulose/hemicellulose. The significance and special qualities of QDs were also emphasized to demonstrate their excellent photocatalytic performance for prospective applications. QD‐based photocatalytic biomass valorization has achieved a great deal and offers a wide range of research opportunities. Whereas the QDs‐based photocatalyst is still facing numerous challenges, the study in this area is still at its initial phase and continuous efforts are required to boost future reformation in photocatalytic biomass valorization on QD‐based catalyst and meet a range of persistent challenges as well as opportunities.

First of all, the photocatalytic biomass conversion of QD‐based photocatalysts doesn't perform satisfactorily at present and the inherent poisonousness of Cd prevents its widespread applications. Future investigation ought to focus on the exploitation of greener, eco‐friendly of QDs with micro‐structure/shape controllable, excited‐state steerability, and surface modifiability. By enhancing absorption and light utilization, manipulating radicals or intermediates to produce desired products, and manufacturing abundant active sites, the catalytic efficiency and product selectivity of biomass conversion in QD‐based photocatalytic systems can be improved. For example, developing or adding cocatalysts with greatly revelational active sites and rapid movement of charge carriers (e.g., MoS_2_, WS_2_) has great potential in enhancing the efficiencies of photocatalytic biomass valorization.^[^ ^97^ ^]^ Besides, QDs also can construct heterostructures with I‐III‐VI_2_ and I_2_‐II‐IV‐VI_4_ semiconductors, which have demonstrated excellent photocatalytic performance in a variety of reactions including biomass valorization.^[^ ^98^ ^]^

Second, QDs‐based catalysts are multicomponent materials with multitudinous organic ligands and functional groups on the catalyst's exterior, which greatly increases the complexity of the photocatalytic systems. There is still a lack of comprehensive and deep understanding about the intermediates and the dynamic changes of the QD‐based photocatalysts during the photocatalytic biomass conversion reaction. Therefore, further study should take more research on intermediates formed and monitor changes in the composition and surface quantivalence status of the QD‐based photocatalyst during the photocatalytic biomass conversion reactions. The investigation focused on surfaces or interfaces may contribute to improve the control of basic reactions. Kevin et al. proved that the selectivity of photocatalytic oxidation into benzyl alcohol benzaldehyde (up to 99%) or C─C coupled products (91%) could be controlled by tuning the amount of low valence nulvalent Cd (Cd^0^) in the developed CdS QDs.^[^ ^99^ ^]^ Time‐course analysis of CdS QDs further provided sufficient information about reaction intermediates and changes of Cd^2+^ that were partially reduced into Cd^0^ on the surfaces. It also provides guidance for the more innovative construction and exploitation of superior photocatalytic biomass conversion based on QDs photocatalysts.

Third, it is difficult to regulate the distribution of the product because of the structural complexity of native biomass containing different amounts of cellulose, hemicellulose, and lignin. Further research should be devoted to develop efficient photocatalysts and reactors for biomass photoreforming, which could stepwise convert functional groups into diverse products, and thus could be easily isolated and extracted. Additionally, it is crucial to research the various transformation mechanisms between native biomass and its model compounds, as these findings may help to guide the photocatalytic transformation of real biomass.

Finally, continuous efforts are needed to explore effective photocatalytic systems that can selectively rupture specific bonds and enhance the selectivity for target products, since it is challenging to prepare a singular product or a group of compounds with alike features in a biomass system. The selectivity of photocatalytic transformation of biomass can be adjusted by changing external conditions. Besides, achieving industrial‐scale application of QD‐based catalysts for photocatalytic biomass valorization remains a distant goal, although several studies have shown it to be a viable method and this concept sounds excellent. But the issues including the entire catalytic separation process, the cost of the catalyst, and whether the catalyst can maintain catalytic activity and stability after scaling up should be considered. There are still many issues to be resolved, and we hope that our work will encourage more research on solar energy‐driven QD‐based photocatalytic biomass valorization.

## CONFLICTS OF INTEREST STATEMENT

The authors declare no conflicts of interest.
